# Estrogen and mechanical loading-related regulation of estrogen receptor-β and apoptosis in tendinopathy

**DOI:** 10.1371/journal.pone.0204603

**Published:** 2018-10-08

**Authors:** Jeng-Long Hsieh, I-Ming Jou, Chao-Liang Wu, Po-Ting Wu, Ai-Li Shiau, Hao-Earn Chong, Yu-Ting Lo, Po-Chuan Shen, Shih-Yao Chen

**Affiliations:** 1 Department of Medical Laboratory Science and Biotechnology, Chung Hwa University of Medical Technology, Tainan, Taiwan; 2 Department of Orthopedics, E-DA Hospital, Kaohsiung, Taiwan; 3 Department of Biochemistry and Molecular Biology, College of Medicine, National Cheng Kung University, Tainan, Taiwan; 4 Department of Orthopedics, College of Medicine, National Cheng Kung University, Tainan, Taiwan; 5 Department of Microbiology and Immunology, College of Medicine, National Cheng Kung University, Tainan, Taiwan; 6 Department of Orthopedics, Tainan Hospital, Ministry of Health and Welfare, Tainan, Taiwan; 7 Department of Nursing, Chung Hwa University of Medical Technology, Tainan, Taiwan; 8 Department of Internal Medicine, College of Medicine, National Cheng Kung University, Tainan, Taiwan; 9 Department of Internal Medicine, National Cheng Kung University Hospital, College of Medicine, National Cheng Kung University, Tainan, Taiwan; Queen Mary University of London, UNITED KINGDOM

## Abstract

Female-dominant tendinopathies are musculoskeletal disorders caused by repetitive hand posture and motion; they are considered overuse syndromes. Both external mechanical stress and changes in hormone levels might affect disease progression. We have previously reported that estrogen receptor-β (ER)-β expression was associated with the pathogenesis of de Quervain's disease. To study the underlying mechanisms, a cyclic stretching culture system was applied to tendon tissue from ovariectomized (OVX) rats. Furthermore, a collagenase I-induced rat tendinopathy model was established to examine the association of ER-β with disease progression. Our results showed that ER-β expression and the number of apoptotic cells were higher and associated with disease severity in rats with tendinopathy. Mechanical stress altered the morphology of primary tenocytes and collagen fiber alignment in tendons, and up-regulated the expression of matrix metalloproteinase-9, ER-β, and interleukin-1β, as well as induced apoptosis in tenocytes and tendon tissue from OVX rats. This is the first report on the effects of ER-β and mechanical stress in tendinopathy. We hope these findings contribute to new pharmacological therapies targeting ER-β signaling pathways to treat tendon-related diseases.

## Introduction

Females are predisposed to develop autoimmune and inflammatory diseases because of sexual dimorphism [1,2]. De Quervain’s disease occurs more frequently in women in the later stages of pregnancy and in early postpartum period than in women who are not pregnant [3,4]. Carpal tunnel syndrome (CTS) is strongly associated with postmenopausal and ovariectomized (OVX) women [5]. Similarly, women older than 53 years are more likely than are men to have trigger finger/digits [6]. Furthermore, pieces of evidence reported the higher risk of tendon rupture in woman with type 2 diabetes and use of quinolones than in men [7,8]. To consider molecular mechanisms, estrogen receptors (ERs) are highly expressed in the transverse carpal ligament and flexor tenosynovium from patients with CTS. ER expression levels in postmenopausal patients are associated with age and are assumed to be related to female-dominant diseases [9]. However, the mechanism through which ERs modulate the pathogenesis of women's tendinopathies remains unclear.

Estrogen is a steroid hormone essential for metabolism and key biological effects, including weight change, food intake, insulin sensitivity, glucose homeostasis, body fat distribution, inflammation, growth, differentiation, and female reproductive tissue development [10,11]. Two major nuclear receptors, ER-α and ER-β, mediate estrogen activity. For instance, estrogen signaling through ER-α is responsible for growth plate fusion in both male and female mice, and ER-β inhibits growth in female mice [12,13]. Moreover, ER-α mediates transforming growth factor-β (TGF-β) signaling and collagen synthesis, and ER-β is involved in cell apoptosis [14,15]. Dysregulated apoptosis has been associated with the pathogenesis of tendon disorders, including rotator cuff [16], Achilles tendon [17], and patellar tendinopathies [18]. Development of apoptosis has also been observed in rat tibialis anterior tendon after high strain mechanical loading [19]. Therefore, it is warranted to analyze the ER-mediated apoptotic process in tendinopathy.

Because ER-β is highly expressed in patients with de Quervain’s disease, ER-β expression levels are assumed to indicate disease severity, which is also associated with tissue inflammation [20]. We hypothesized that ER-β expression was involved in the pathogenesis of women's tendinopathies. This study was undertaken to examine the role of ER-β in tendinopathy using a dynamic cyclic stretching culture system to culture tendon tissue and tenocytes. This culture system provides a closer look at how the cells respond during repetitive motion. Furthermore, we analyzed OVX rats whose Achilles tendon were isolated for dynamic cyclic stretching to determine the effects of mechanical loading and estrogen deficiency on tendinopathy.

## Materials and methods

### Ethics statement

All experimental rats were purchased from the Animal Center at National Cheng Kung University, and the animal experiments were done strictly in accordance with protocols approved by the Institutional Animal Care and Use Committee of National Cheng Kung University.

### Animal model

Rat tendinopathy model was used as described in detail previously [21]. Briefly, adult female Sprague-Dawley rats (250–300 g) were anesthetized with isoflurane and the hair on their lower limbs was shaved to prevent signal disturbance. An ultrasound transducer in a hands-free stand 4.5 mm above the center of the tendon captured images taken perpendicular to the Achilles tendon. The axis of the linear transducer was aligned along the long axis of the Achilles tendon. Using real-time ultrasonic guidance, the needle was placed parallel to the long axis of the transducer to confirm that the injection was peritendinous. To induce tendinopathy, the rats’ right Achilles tendons were intratendinously injected (ultrasonography-guided) with collagenase I (0.015 mg/ml) (Sigma-Aldrich, St. Louis, MO) to induce tendinopathy. The contralateral left leg was injected with phosphate-buffered saline (PBS). An ovariectomy was performed. Two weeks after ovariectomy, the tendon tissue from rats were isolated and subjected to dynamic stretching for 24h.

### Cell and tissue dynamic cyclic stretching system

Isolated tendon tissue from normal and OVX rats and rat primary tenocytes were loaded and cultured in a dynamic cyclic stretching system (ATMS Boxer^TM^, TAIHOYA Corp., Kaohsiung, Taiwan). The system consisted of rectangular, elastic silicon chambers in which the whole dish, not only the cell culture surface, was deformable (S1 Fig). The dishes were designed to be used in a stimulation apparatus driven by an eccentric motor that allows variation in the amplitude (5–15%) and frequency (0.5–2.0 Hz) of applied strain (S2 Fig). Primary tenocytes were seeded (1 x 10^5^ cells per well) in the chambers coated with type I collagen. Mechanical loading was used according to the pilot studies (amplitude: 15%, frequency: 1 Hz, time: 24h) based on observing the optimized number of apoptotic cells. Cells were treated with anti-ER-β (ab3576, Abcam) and isotype control antibodies (4 μg/ml) and followed by cyclic stretching as described above.

#### Histological and immunohistochemical analyses

The mice were killed, and theircollagenase I-treated tendon tissue were fixed with 4% paraformaldehyde, and stained with hematoxylin & eosin to evaluate the change of collagen alignment. Based on our previous study described in detail [22], a modified semiquantitative 4-point scoring method for each factor was used. Tendinopathy severity, based on the sum of the scores, was graded as 0–3 (0, ≤ 2, 3–4, ≥5 points). After scoring, the tendon tissue were subjected to undergo immunohistochemistry and TUNEL assay. The tendon tissue from normal and overiectomy rats (under static and stretch conditions) were immunohistochemically analyzed. The processed tissuewere snap-frozen and embedded in paraffin. The sections were deparaffinized in xylene, dehydrated in alcohol, treated with proteinase K, washed with H_2_O_2_ in PBS, and stained with antibodies against ER-β (Abcam), collagen type I (Abcam), cleaved caspase 3 (Cell Signaling Technology), interleukin-1β (IL)-1β (Cell Signaling), and matrix metalloproteinase-9 (MMP)-9 (Abcam), in combination with the chromogen 3-amino-9-ethylcarbazole (Zymed). The signal intensity was further quantitated using Image J 1.42q (National Institutes of Health) in three randomly chosen fields.

#### Primary culture of rat tenocyte

Tenocytes were isolated from the Achilles tendons, cultured and identified using anti-tenomodulin antibody (Santa Cruz) as described in detail previously [23].

#### Immunofluorescence and TUNEL assessments

The processed tenocytes were immunofluorescence stained with antibodies against ER-β, IL-1β, MMP-9, and α-tubulin, followed by Alexa Fluor 488- and Alexa Fluor 594-conjugated secondary antibodies (Life Technologies), respectively. The DeadEnd colorimetric terminal deoxynucleotidyl transferase-mediated dUTP nick end labeling (TUNEL) assay kit (Promega) was used to detect apoptotic cells in the tendon tissue and tenocytes.

#### Statistical analysis

Data are expressed as mean ± standard error of the mean (SEM). Statistical significance among multiple groups was assessed using analysis of variance (ANOVA) with Dunnett's adjustment for multiple comparisons. The correlations among ER-β expression, the number of TUNEL-positive cells and grade of tendinopathy were analyzed using the Spearman correlation rank test. P values less than 0.05 were considered significant.

## Results

### Increased ER-β levels and enhanced apoptosis in tendon tissue from rats with tendinopathy

**Fig 1:**
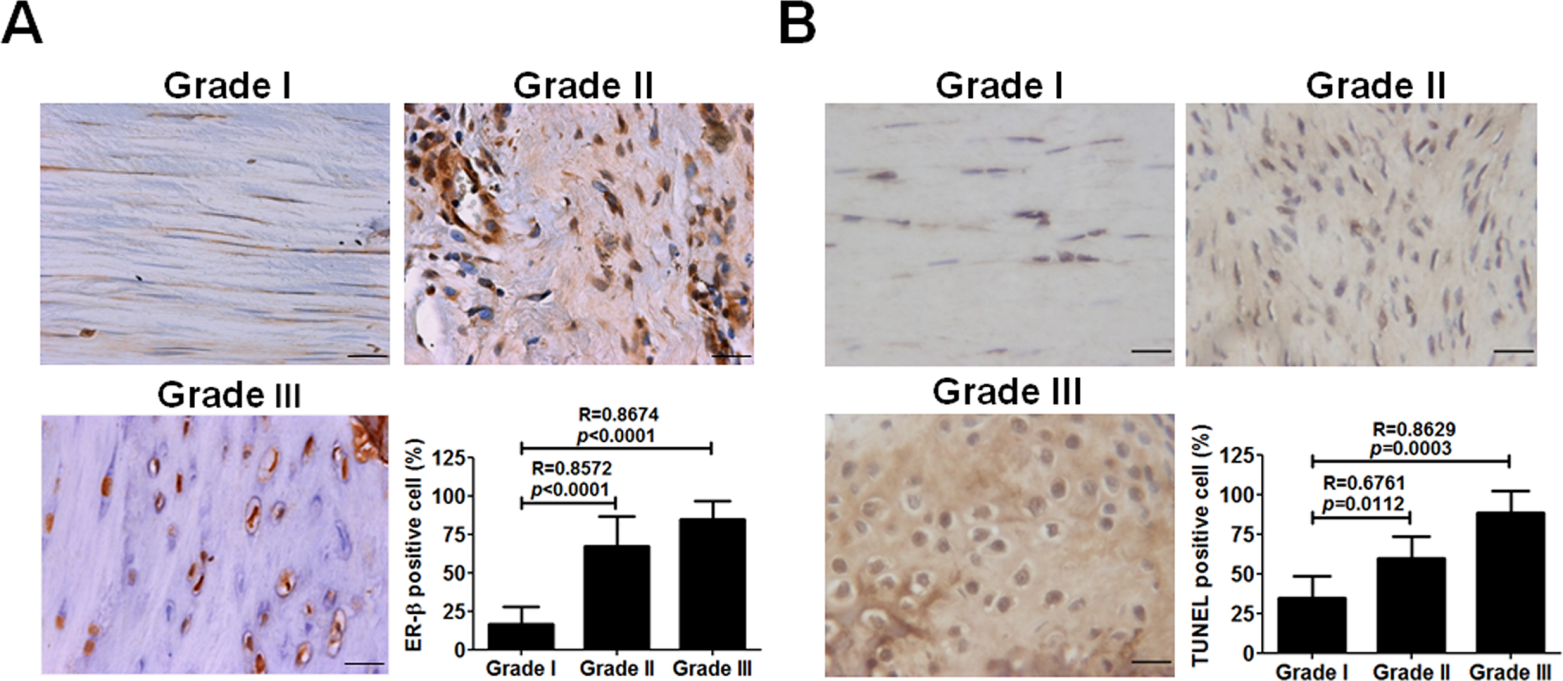
Estrogen receptor-β (ER-β) expression and apoptosis in tendon tissue from rats with tendinopathy. Adult female Sprague-Dawley rats (200~300 g) were treated with ultrasound-guided intra-tendinous injection of collagenase I into their Achilles tendons for 1,4, and 8 weeks. **A,** Immunohistochemical staining of ER-β and the number of ER-β-positive cells (n = 15). **B,** TUNEL staining and the number of TUNEL-positive cells (n = 5~8) in tendon tissue from rats with different grades of tendinopathy. Scale bars represent 50 μm in × 400 magnifications. Data are mean ± standard error of the mean (SEM). R represents Spearman correlation coefficients.

Because ER-β expression is positively correlated with the histological grade of de Quervain’s disease [20], to assess its role in the animal model of tendinopathy, we examined the ER-β expression in the Achilles tendon from tendinopathic rats. Immunohistochemical staining and TUNEL assays showed that ER-β and apoptotic cells were detected with increased levels and number and associated with the histological grade during progression of tendinopathy (Fig 1A and 1B).

### Increased expression of ER-β, IL-1β, MMP-9, and enhanced apoptosis in tenocytes after high-magnitude mechanical strain

**Fig 2:**
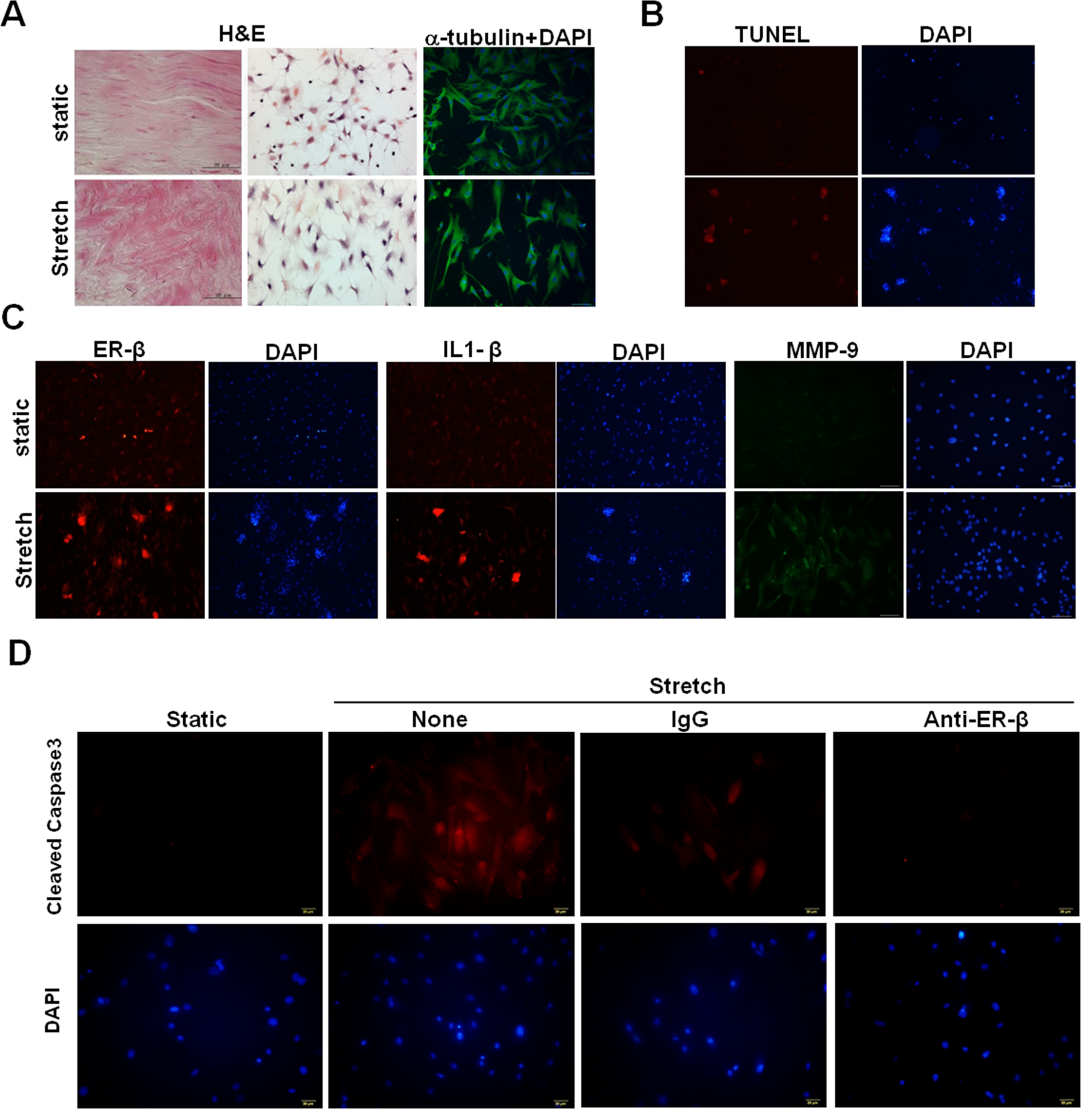
ER-β, interleukin (IL)-1β, matrix metalloproteinase (MMP)-9 expression and apoptosis in cyclically stretched rat primary tenocytes. **A,** Representative morphological images of static and mechanically strained tissue (left) and cultured primary tenocytes (middle), as determined by hematoxylin and eosin (H&E) staining. Scale bars represent 100 μm in × 200 magnifications. Expression of α-tubulin (right) in tenocytes after cyclic stretching, as determined by immunofluorescence staining. **B,** TUNEL assay in tenocytes after cyclic stretching. Nuclei were counterstained with DAPI. **C,** ER-β, IL-1β, and MMP-9 expression in tenocytes after cyclic stretching, as determined by immunofluorescence staining. **D,** Cleaved caspase 3 expression in anti-ER-β and isotype control antibody (IgG)-treated tenocytes during cyclic stretching for 24h, as determined by immunofluorescence staining.

We further used a dynamic cyclical culturing system on rat primary tenocytes to mimic clinical manifestations. Compared with cells cultured on static silicon surfaces, tenocytes became rounder and flatter after they had been cyclically stretched for 24h (Fig 2A, middle panels). However, α-tubulin expression levels did not change (Fig 2A, right panels). At the tissue level, the alignment of fiber bundles twisted under cyclic tensile stretch when compared to unstretched tissue (Fig 2A, left panels). TUNEL assays showed that enhanced apoptosis were observed in mechanically stretched tenocytes compared with static cultured controls (Fig 2B). Furthermore, immunofluorescence staining showed that ER-β, IL-1β, and MMP-9 expression levels were higher in stretched tenocytes than in statically cultured cells (Fig 2C). Cleaved caspase 3 expression was reduced in tenocytes treated with a neutralizing antibody against ER-β during cyclic stretching (Fig 2D), implicating an ER-β-mediated mechanism might be involved in mechanical loading-induced apoptosis.

### Regulation of ER-β expression and apoptosis in tendon tissue by estrogen and cyclic tensile stretch

**Fig 3:**
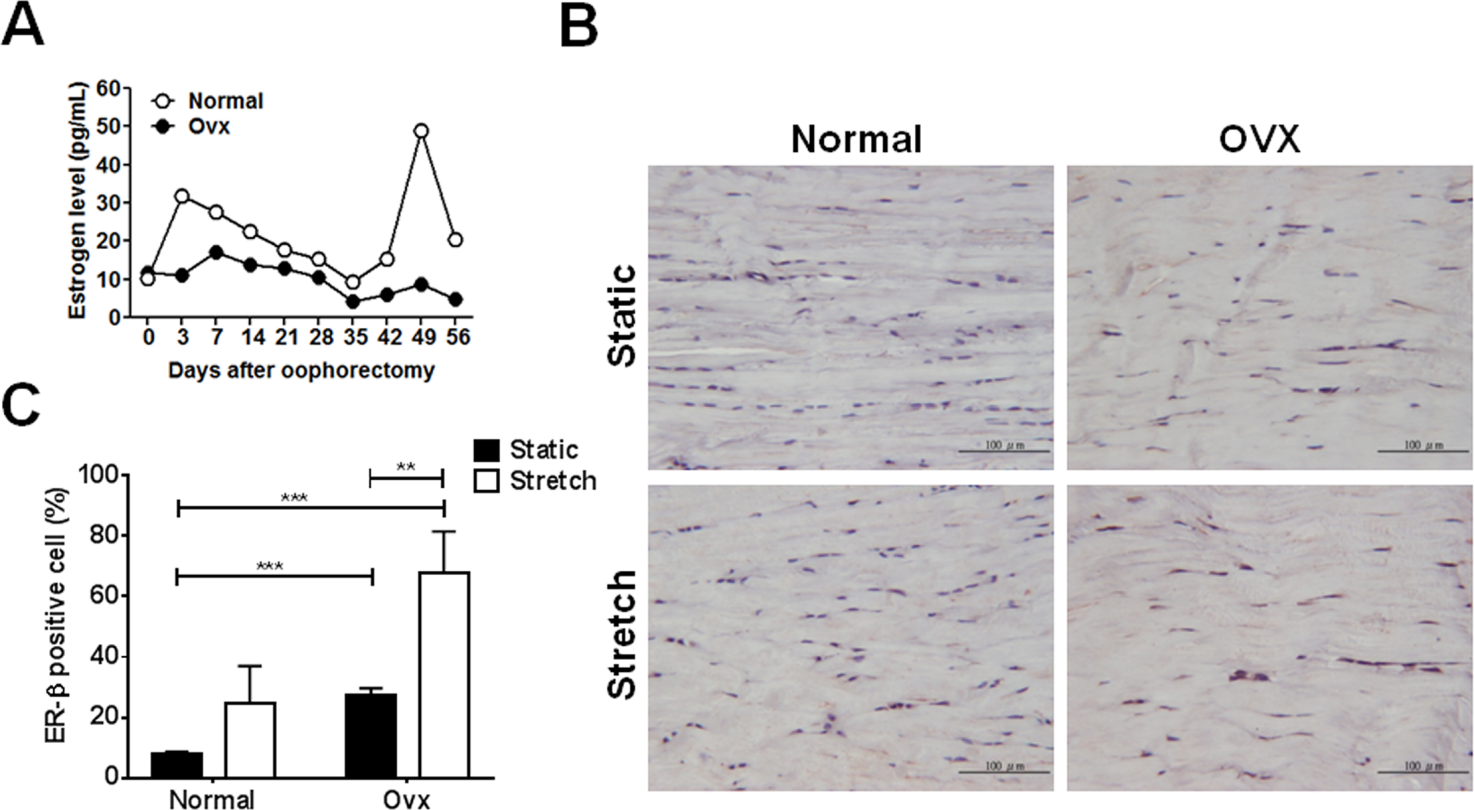
Expression of ER-β in tendon tissue from ovariectomized rats after cyclic stretching. An ovariectomy in adult female Sprague-Dawley rats (200~300 g) was performed and their Achilles tendons were isolated and applied to the following stretch protocol: 15%, 2Hz, time period: 24h **A,** Serum samples were collected every other day and determined the estrogen levels by ELISA. Ovx, rats were performed with ovariectomy. **B,** Expression of ER-β and **C,** calculated ER-β-positive cells in tendon tissue from ovariectomized rats with and without cyclic stretching. Data are mean ± SEM (n = 3). **p<0.01, ***p<0.001.

Estrogen levels started to fall 3 days after ovariectomy and stabilized 35 days after ovariectomy in female rats (Fig 3A). Compared to control tendon from normal rats under static condition, the expression of ER-β was up-regulated in rats 21 days after ovariectomy. In particular, ER-β expression was even higher in the stretched tendons from ovariectomized rats than the other three groups, as determined by immunohistochemical staining (Fig 3B and 3C).

**Fig 4:**
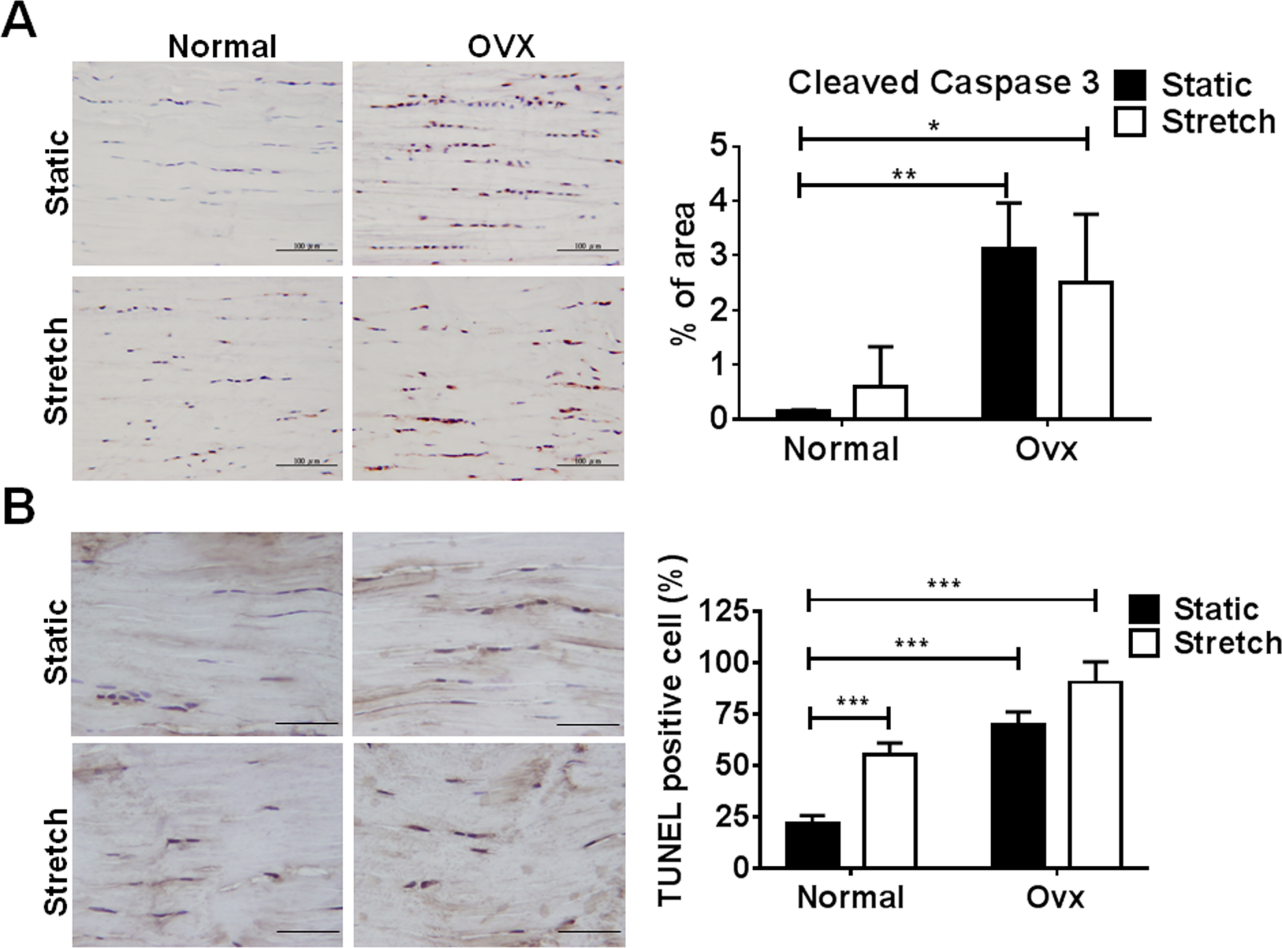
Cleaved caspase 3 expression and apoptosis in tendon tissue from ovariectomized rats with and without cyclic stretching. **A,** Immunohistochemical staining of cleaved caspase 3 and calculated cleaved caspase 3-expressed areas. **B,** TUNEL staining and the number of TUNEL-positive cells in tendon tissue from ovariectomized rats after cyclic stretching. Scale bars represent 100 and 50 μm in × 200 and × 400 magnifications. Values are the mean ± SEM (n = 3). *p<0.05, **p<0.01, ***p<0.001.

To evaluate the effects of increased ER-β expression on cell apoptosis, TUNEL assay and immunohistochemical staining of active caspase 3 were applied to tendon tissue suffering from estrogen deficiency and cyclic tensile stretching. The expression levels of cleaved caspase 3 and apoptotic cell number were higher in tendon tissue from ovariectomized rats than in those from normal rats under both static and stretched conditions (Fig 4A and 4B).

### Regulation of type I collagen (COL1), IL-1β, and MMP-9 expression in tendon tissue by estrogen and cyclic tensile stretch

**Fig 5:**
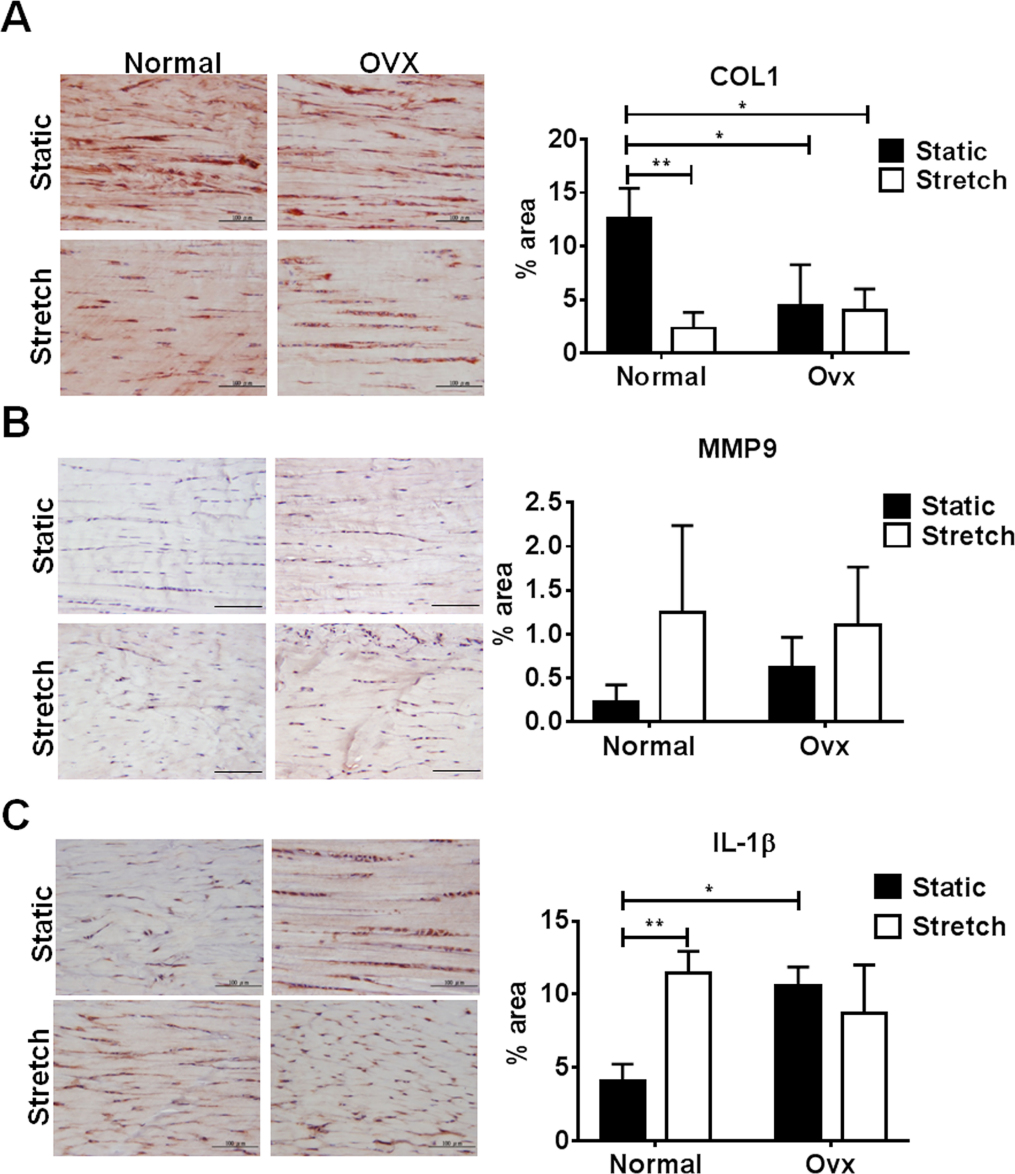
Expression of collagen type I (COLI), matrix metalloproteinase (MMP)-9, and IL-1β in tendon tissue from ovariectomized rats after cyclic stretching. **A,** Immunohistochemical staining of COLI and calculated COLI-expressed area **B,** MMP-9 and calculated MMP-9-expressed area and **C,** IL-1β and calculated IL-1β-expressed area in tendon tissue from ovariectomized rats after cyclic stretching. Scale bars represent 100 μm in ×200 magnifications. Values are SEM (n = 3). *p<0.05, **p<0.01.

Because collagen switch from type I to III is a key early phenotypic change in tendinopathy which results in biomechanical inferiority and degeneration [24]. Furthermore, observations from unloaded healing rat flexor tendons have demonstrated that MMP-9 mediate tissue degradation during the early phase of healing [25]. Therefore, we examined the expression of COL1, IL-1β, and MMP-9 in estrogen deficient tendon tissue under cyclic tensile stretching. Decreased COL1 expression was observed in tendon tissue from ovariectomized rats under both static and stretched conditions, as well as those from normal rats under stretched condition when compared to normal static cultured cells (Fig 5A). In contrast, increased expression of MMP-9 and IL-1β was observed in tendon tissue from ovariectomized rats under both static and stretched conditions, as well as those from normal rats under stretched condition when compared to normal static cultured tendons (Fig 5B and 5C). Taken together, these results indicate that mechanical loading might synergize with estrogen deficiency to affect the expression of ER-β and the downstream pathological effects in tendon diseases.

## Discussion

De Quervain’s disease, CTS, and trigger finger/digits occur predominantly in females and are related to age and specific occupations. Therefore, simultaneous endogenous hormone changes and exogenously induced mechanical stress might mutually affect disease progression. High level mechanical strain of articular chondrocytes induces phenotypical changes associated with osteoarthritis and pain [26]. However, the effects of changing the mechanical loading on tenocyte metabolism are unclear. Lack of evidence considering endogenous and exogenous factors that affect hand tendinopathies make it difficult to understand disease pathogenesis. Therefore, a dynamic cyclic stretching culture system might partially resolve the puzzle. To our knowledge, this study is the first to reveal the effects of ER-β and mechanical loading on tendinopathy, including their associations with tenocyte apoptosis (Fig 1 and Fig 2). These results suggested that cyclic tensile stretching could synergize with estrogen defect to enhance ER-β gene expression.

The proinflammatory cytokine IL-1β has been considered as an initiator of tendinopathy. It can be released by tenocytes in response to mechanical loading, resulting in the production of MMPs [27,28]. It can also inhibit the production of type I collagen in tendon-derived cells [29]. According to our findings, mechanical strain and estrogen deficiency down-regulate the expression of COL1, but up-regulates the expression of IL-1β and MMP-9 in tendon tissue and tenocytes (Fig 2 and Fig 5). A study on healing rat flexor tendons suggests that MMP-9 and MMP-13 mediate COL1 degradation during the early phase of healing [25]. Therefore, a positive feedback loop might exist as an IL-1β-mediated mechanism through mechanical loading in tendinopathy, and ER-mediated signaling pathways might also be involved.

Our study has some limitations. Firstly, we observed that mechanical loading and estrogen deficiency had additive effects on changes in the gene expression of ER-β (Fig 3), but not on their downstream pathological effects (Fig 4 and Fig 5). We hypothesize that the ER-α and -β-mediated signaling pathways might have contradictory roles in tendinopathy. For instance, impaired development of cartilage can be observed in ER-α null mice since the crosstalk between ER-α and TGF-β1 pathways is responsible for the expression of type II collagen in primary chondrocytes [14]. Estrogen signaling through the ER-β pathway impairs cartilage growth and type II collagen expression [30]. Therefore, knockout or knockdown systems for ER-α and -β are required to clearly examine their roles in tendinopathy. For instance, lentivirus-based short-hairpin RNA or CRISPR/Cas9 targeting either ER-α or -β can be delivered into Achilles tendon from rats with tendinopathy by ultrasound-guided intra-tendinous injection. The knockdown or knockout efficiency of ER-α and -β will further be correlated with the tendinopathic signs to assign detailed mechanisms to our present work. Secondly, a rat Achilles tendinopathy used to model hand diseases here might not be suitable since known knowledge demonstrated that Achilles tendon rupture rates were widely and thoroughly acknowledged to be strongly male dominant. USA health care data indicates males sustained ruptures more frequently than females (male versus female; 141 per 100,000 versus 67 per 100,000) [31]. In a 10-year-period in Sweden of 27,702 patients with acute Achilles tendon ruptures 79% were men (21,979 men) [32]. According to previous epidemiology studies, hand abnormalities (de Quervain’s, carpal tunnel syndrome and trigger finger) are related to gender and certain occupations [6,33–35]. We suggested these tendon abnormalities would have substantial proportion in population in addition to exercise-induced tendon injuries. Based on a population-base study, the prevalent rate of above mentioned hand diseases is 2.2% in Taiwan (personal communication). Therefore, estrogen (gender) and mechanical loading (occupation) may both affect disease pathogenesis. Furthermore, ER-independent signaling pathways may not be ruled out as disease regulators, which require further investigations to address these issues.

In conclusion, we found, by analyzing the tendon tissue and tenocytes from rats, that mechanical loading and estrogen deficiency exacerbated the disease through up-regulating ER-β level and inducing apoptosis. ER-β-associated apoptosis is crucial for the pathogenesis of tendinopathy. These findings might contribute to developing novel pharmacological therapies that target ER-β signaling pathways in tendon-related diseases.

## Supporting information

S1 Fig Flexible silicone dishes used for cultivation and strain. The rectangular dishes are 60 mm long × 20 mm wide ×3 mm high, and the wells have a 20 mm × 20 mm cell culture surface.
(TIF)

S2 Fig The ATMS Boxer^TM^ dynamic stretching culture system. The device is comprised of an electrical control with the maximum of six sets of silicon membrane.
(TIF)
