# Supplementary figures and images for: Estrogen and mechanical loading-related regulation of estrogen receptor-β and apoptosis in tendinopathy
Source: PLoS One. 2018 Oct 8;13(10):e0204603. doi: 10.1371/journal.pone.0204603 (PMC6175495; doi:10.1371/journal.pone.0204603)

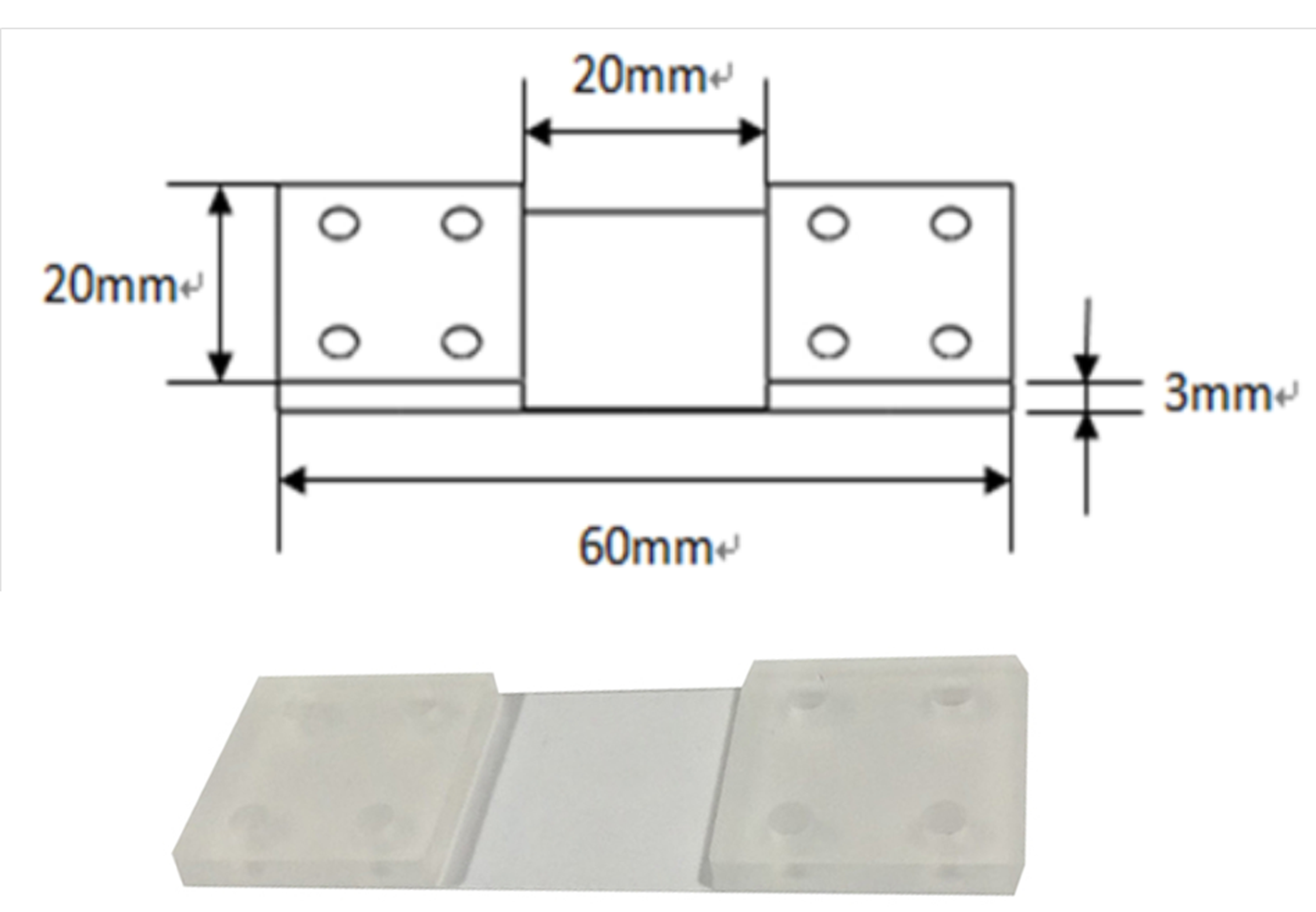

Supplement: S1 Fig — The rectangular dishes are 60 mm long × 20 mm wide ×3 mm high, and the wells have a 20 mm × 20 mm cell culture surface. (TIF) [file pone.0204603.s001.tif]

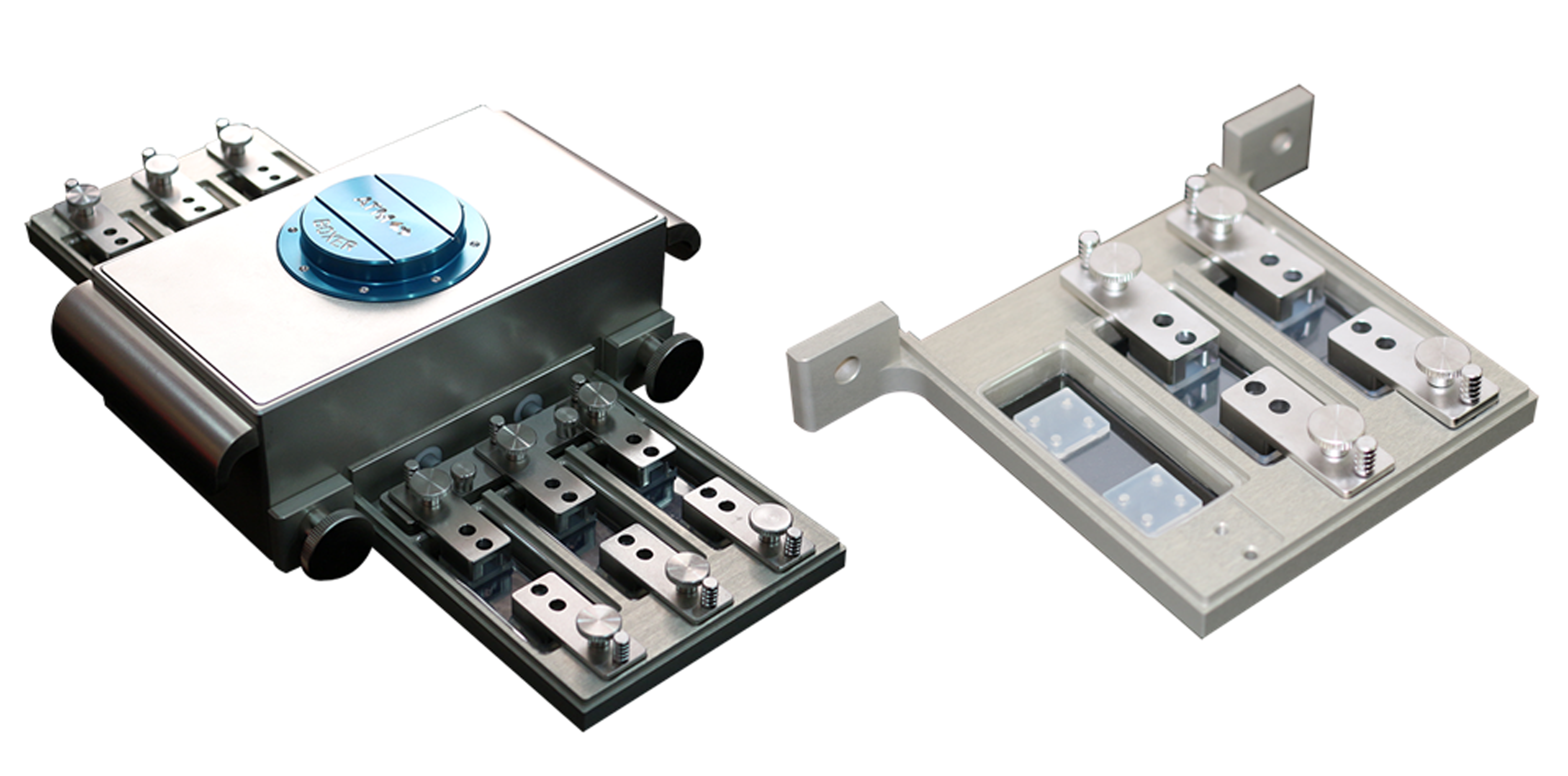

Supplement: S2 Fig — The device is comprised of an electrical control with the maximum of six sets of silicon membrane. (TIF) [file pone.0204603.s002.tif]
